# Pathogenicity and Transcriptomic Analyses of Two “*Candidatus* Liberibacter asiaticus” Strains Harboring Different Types of Phages

**DOI:** 10.1128/spectrum.00754-23

**Published:** 2023-04-18

**Authors:** Yongqin Zheng, Jingxue Zhang, Yun Li, Yaoxin Liu, Jiayin Liang, Cheng Wang, Fang Fang, Xiaoling Deng, Zheng Zheng

**Affiliations:** a National Key Laboratory of Green Pesticide, South China Agricultural University, Guangzhou, Guangdong, China; b Guangdong Province Key Laboratory of Microbial Signals and Disease Control, South China Agricultural University, Guangzhou, Guangdong, China; c Horticulture Research Institute, Guangxi Academy of Agricultural Sciences, Nanning, Guangxi, China; USDA—San Joaquin Valley Agricultural Sciences Center

**Keywords:** “*Candidatus* Liberibacter asiaticus”, phage, *Catharanthus roseus*, pathogenicity difference, transcriptomic analysis

## Abstract

“*Candidatus* Liberibacter asiaticus” is one of the putative causal agents of citrus Huanglongbing (HLB), a highly destructive disease threatening the global citrus industry. Several types of phages had been identified in “*Ca*. Liberibacter asiaticus” strains and found to affect the biology of “*Ca*. Liberibacter asiaticus.” However, little is known about the influence of phages in “*Ca*. Liberibacter asiaticus” pathogenicity. In this study, two “*Ca*. Liberibacter asiaticus” strains, PYN and PGD, harboring different types of phages were collected and used for pathogenicity analysis in periwinkle (Catharanthus roseus). Strain PYN carries a type 1 phage (P-YN-1), and PGD harbors a type 2 phage (P-GD-2). Compared to strain PYN, strain PGD exhibited a faster reproduction rate and higher virulence in periwinkle: leaf symptoms appeared earlier, and there was a stronger inhibition in the growth of new flush. Estimation of phage copy numbers by type-specific PCR indicated that there are multiple copies of phage P-YN-1 in strain PYN, while strain PGD carries only a single copy of phage P-GD-2. Genome-wide gene expression profiling revealed the lytic activity of P-YN-1 phage, as evidenced by the unique expression of genes involved in lytic cycle, which may limit the propagation of strain PYN and lead to a delayed infection in periwinkle. However, the activation of genes involved in lysogenic conversion of phage P-GD-1 indicated it could reside within the “*Ca*. Liberibacter asiaticus” genome as a prophage form in strain PGD. Comparative transcriptome analysis showed that the significant differences in expression of virulence factor genes, including genes associated with pathogenic effectors, transcriptional factors, the Znu transport system, and the heme biosynthesis pathway, could be another major determinant of virulence variation between two “*Ca*. Liberibacter asiaticus” strains. This study expanded our knowledge of “*Ca*. Liberibacter asiaticus” pathogenicity and provided new insights into the differences in pathogenicity between “*Ca*. Liberibacter asiaticus” strains.

**IMPORTANCE** Citrus Huanglongbing (HLB), also called citrus greening disease, is a highly destructive disease threatening citrus production worldwide. “*Candidatus* Liberibacter asiaticus” is one of the most common putative causal agents of HLB. Phages of “*Ca*. Liberibacter asiaticus” have recently been identified and found to affect “*Ca*. Liberibacter asiaticus” biology. Here, we found that “*Ca*. Liberibacter asiaticus” strains harboring different types of phages (type 1 or type 2) showed different levels of pathogenicity and multiplication patterns in the periwinkle plant (Catharanthus roseus). Transcriptome analysis revealed the possible lytic activity of type 1 phage in a “*Ca*. Liberibacter asiaticus” strain, which could limit the propagation of “*Ca*. Liberibacter asiaticus” and lead to the delayed infection in periwinkle. The heterogeneity in the transcriptome profiles, particularly the significant differences in expression of virulence factors genes, could be another major determinant of difference in virulence observed between the two “*Ca*. Liberibacter asiaticus” strains. These findings improved our understanding of “*Ca*. Liberibacter asiaticus”-phage interaction and provided insight into “*Ca*. Liberibacter asiaticus” pathogenicity.

## INTRODUCTION

The phloem-limited bacterium “*Candidatus* Liberibacter asiaticus” is the causal agent of citrus Huanglongbing (HLB), also called citrus greening disease, one of the most destructive citrus diseases worldwide (1, 2). “*Ca*. Liberibacter asiaticus” can infect nearly all commercial citrus cultivars and cause diverse symptoms, such as leaf mottling, yellowing shoots, Zn-deficiency-like symptoms, uneven coloring of fruits, etc. (2). However, the long latency period and disease process of HLB in the citrus host and the inability to culture “*Ca*. Liberibacter asiaticus” *in vitro* make “*Ca*. Liberibacter asiaticus” and HLB research more challenging. Alternatively, “*Ca*. Liberibacter asiaticus” can be transmitted to a nonnatural host—e.g., periwinkle (Catharanthus roseus) (3, 4), tomato (Solanum lycopersicum), and tobacco (Nicotiana tabacum) (5, 6)—via the parasitic dodder plant (*Cuscuta* spp.). Among these nonnatural hosts, periwinkle was found to be a more amenable and suitable host for “*Ca*. Liberibacter asiaticus” research, mainly because of its abilities at rapid establishment and multiplication of “*Ca*. Liberibacter asiaticus” and shortening the latency to development of symptoms within 2 months after inoculation with “*Ca*. Liberibacter asiaticus” (7, 8). From this, periwinkle has been employed as a surrogate host for “*Ca*. Liberibacter asiaticus” research in several aspects, such as taxonomic characterization of “*Ca*. Liberibacter asiaticus” strains (9), *in planta* distribution pattern analysis of “*Ca*. Liberibacter asiaticus” (8), screening of chemical compounds for “*Ca*. Liberibacter asiaticus” control (10, 11), “*Ca*. Liberibacter asiaticus” genomic DNA enrichment (7, 12, 13), screening of “*Ca*. Liberibacter asiaticus” pathogenic effectors (14), and analyses of “*Ca*. Liberibacter asiaticus”-host interaction (15, 16).

Advances in high-throughput sequencing technology have greatly facilitated the research on “*Ca*. Liberibacter asiaticus” biology, particularly the discoveries of “*Ca*. Liberibacter asiaticus” phages (12, 17, 18). Two types of “*Ca*. Liberibacter asiaticus”-associated phages, SC1 (type 1) and SC2 (type 2), were initially identified in the “*Ca*. Liberibacter asiaticus” strain UF506 through metagenomic analysis (12). SC1 carried suspected lytic cycle genes and could exist as a lytic form *in planta*, while SC2 lacked the lytic cycle genes and was involved in lysogenic conversion. Later, a third type of prophage, P-JXGC-3 (type 3), which carried a restriction-modification (R-M) system, was identified in “*Ca*. Liberibacter asiaticus” strain JXGC from China (17). Most recently, a novel *Microviridae* phage (CLasMV1) with a small circular genome (~8.8 kb) was identified in “*Ca*. Liberibacter asiaticus” strains and found to be widely distributed in a “*Ca*. Liberibacter asiaticus” population from China (18).

Research on “*Ca*. Liberibacter asiaticus” phages/prophages has revealed their critical roles in the genomic evolution and biology of “*Ca*. Liberibacter asiaticus.” As one of the most dynamic components in the “*Ca*. Liberibacter asiaticus” genome, the phage/prophage loci have been used for differentiation of “*Ca*. Liberibacter asiaticus” strains from different geographical locations, including China (19–22), the United States (23, 24), Brazil (25), and Thailand (26). In China, “*Ca*. Liberibacter asiaticus” strains that harbored a single type 2 prophage predominated in “*Ca*. Liberibacter asiaticus” populations from low-altitude regions (<500 m above sea level, mainly including Guangdong and its neighboring provinces), while in “*Ca*. Liberibacter asiaticus” populations from high-altitude regions (>1,000 m above sea level, including Yunnan, Sichuan and Guizhou provinces) either “*Ca*. Liberibacter asiaticus” strains harboring a type 1 prophage or “*Ca*. Liberibacter asiaticus” containing both type 1 and type 3 prophages predominated (21, 22). In addition, genes carried by “*Ca*. Liberibacter asiaticus” phages/prophages were found to play important roles in pathogenicity, adaptability, and survival of “*Ca*. Liberibacter asiaticus” in its host (14, 27–29). The SC1 phage has holin (SC1_gp110) and endolysin genes (SC1_gp035), which may participate in limiting the growth and host range of “*Ca*. Liberibacter asiaticus” (27). The SC2 prophage encodes a peroxidase (SC2_gp095), which is able to inhibit the reactive oxygen-mediated host defenses induced by “*Ca*. Liberibacter asiaticus” infection (14). Overexpression of a prophage-carried gene (LasP235, a homologue of SC1_gp235) induced HLB-like symptoms and altered biosynthesis of secondary metabolite pathways in Carrizo citrange (Citrus sinensis × Poncirus trifoliata) (28). In addition, a nonclassical “*Ca*. Liberibacter asiaticus” prophage-encoded secretory protein (AGH17470, a homologue of SC2_gp205) was found to induce a strong immune response by upregulating the pathogenesis-related genes and promoting salicylic acid (SA) accumulation in citrus plants (29). Although genes carried by different types of phages/prophages had been proven to influence “*Ca*. Liberibacter asiaticus” pathogenicity, whether “*Ca*. Liberibacter asiaticus” strains harboring different types of phages/prophages exhibit differences in pathogenicity during plant infection remains unclear.

In our recent study, single infection of “*Ca*. Liberibacter asiaticus” strains harboring different types of phages (type 1 or type 2) was found to cause distinct HLB symptoms in Citrus reticulata *Blanco* cv. ‘Nianju’ (30). To further evaluate the pathogenicity of “*Ca*. Liberibacter asiaticus” strains with different types of phages, two “*Ca*. Liberibacter asiaticus” strains (PGD and PYN) were collected and maintained in periwinkle (Catharanthus roseus) via an inoculation assay. Strain PYN (originally from Yunnan Province) contained the type 1 phage, and strain PGD (originally from Guangdong Province) contained the type 2 phage. The symptom development and bacterial titer of periwinkle inoculated with each “*Ca*. Liberibacter asiaticus” strain were regularly monitored after inoculation. Our results showed a higher virulence of strain PGD than strain PYN, which exhibited a more severe symptom and rapid replication of bacterial cells in the infected periwinkle plant. Global gene expression profiles of two “*Ca*. Liberibacter asiaticus” strains in periwinkle during infection were investigated and compared to reveal the “*Ca*. Liberibacter asiaticus”-phage interaction and characterize the expression of genes involved in virulence variation between two strains. This study not only expanded our knowledge of difference in pathogenicities between “*Ca*. Liberibacter asiaticus” strains, but also provided new insights into the pathogenesis of “*Ca*. Liberibacter asiaticus” and “*Ca*. Liberibacter asiaticus”-phage interaction.

## RESULTS AND DISCUSSION

### Symptom development of periwinkle plant infected with strain PGD or PYN.

**FIG 1:**
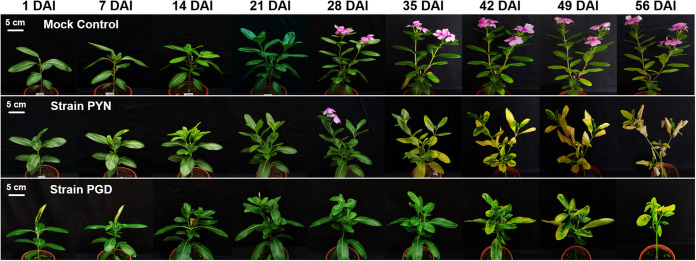
Symptom development on periwinkle (Catharanthus roseus) plants inoculated with different strains of “*Candidatus* Liberibacter asiaticus.” Shown are representative photos of the 15 periwinkle plants used for each condition: plants infected with strain PYN, plants infected with strain PGD, and mock control plants (grafted with a leaf from “*Ca*. Liberibacter asiaticus”-free periwinkle). DAI, days after inoculation.

Distinct differences in symptom development were observed between periwinkle plants infected solely with “*Ca*. Liberibacter asiaticus” strain PGD versus those infected solely with strain PYN after inoculation, especially in the symptoms related to leaf growth and new flush. A smaller leaf was observed in the new flush of the strain PGD-infected periwinkle plant at 14 days after inoculation (DAI), while no obvious symptom was found in strain PYN-infected periwinkle at 14 DAI (Fig. 1). At 35 DAI, the yellowing of mesophyll began on the top leaf of new flush in the strain PGD-infected periwinkle plant, while the obvious yellowing symptoms was observed in both top leaves and mature leaves at 35 DAI. The mottling or yellowing leaf symptom rapidly spread to other parts of the infected plants in both strain PGD- and PYN-infected periwinkle after 35 DAI (Fig. 1). All leaves in periwinkle plants infected by “*Ca*. Liberibacter asiaticus” strain PGD or PYN showed mottled/yellowing symptoms at 42 DAI (Fig. 1). The leaf defoliation was observed at 49 DAI in both strain PGD-infected and strain PYN-infected periwinkle plants.

**FIG 2:**
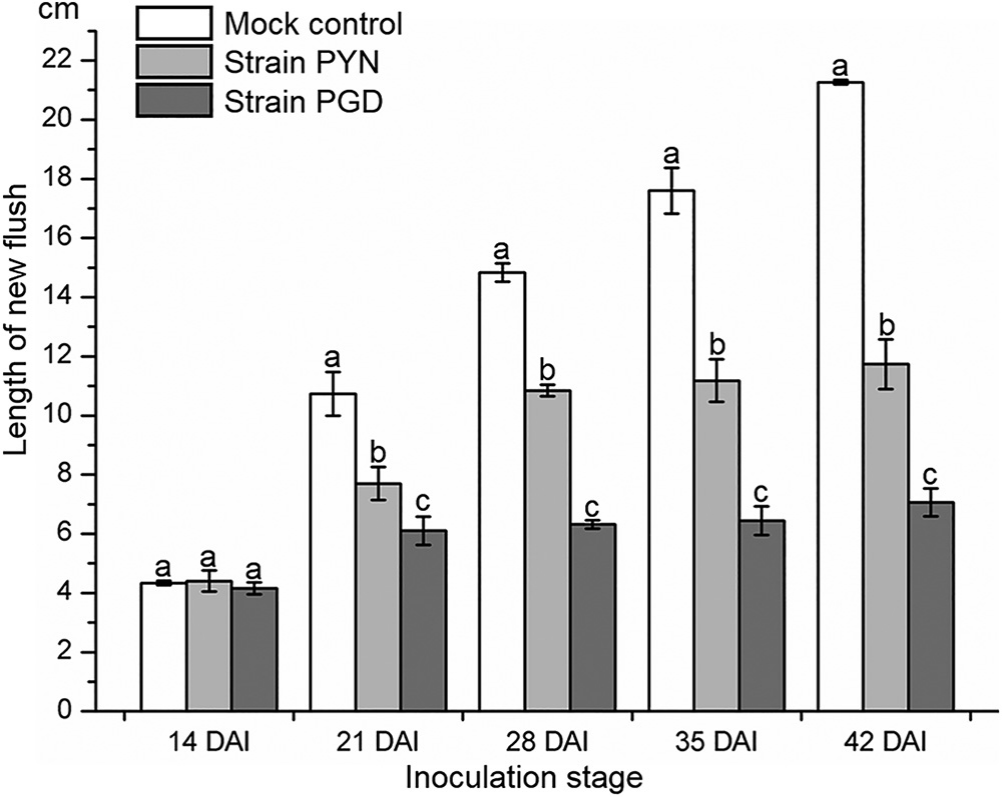
Lengths of newly grown flushes on periwinkle (Catharanthus roseus) with or without “*Candidatus* Liberibacter asiaticus” infection. DAI, days after inoculation. The mock control was grafted with a leaf from “*Ca*. Liberibacter asiaticus”-free periwinkle. The different letters at the top of the bar represent significant difference by single-factor analysis of variance (Duncan’s multiple-range test) at a 95% (*P* < 0.05) confidence interval.

Compared to the mock-grafted control, both strain PGD- and PYN-infected plants exhibited dwarfing symptoms after 14 DAI (Fig. 1). Particularly, the growth of new flush was severely inhibited in the “*Ca*. Liberibacter asiaticus”-infected plants (Fig. 2). At 21 DAI, the infected plants had shorter shoots than the control group (Fig. 2). However, the average length of new flush in strain PGD-infected periwinkle was significantly shorter than those observed in strain PYN-infected periwinkle at 21 DAI (*P* < 0.05), indicating more severe inhibition of the growth of new flush by strain PGD than strain PYN at early infection stage (Fig. 2). In addition, there was nearly no growth of new flush in strain PGD-infected periwinkle after 21 DAI, while a continued growth of new flush was still observed in strain PYN-infected periwinkle between 21 and 28 DAI (Fig. 2). The new flush of mock-grafted periwinkle plants kept growing during the entire observational period (from 14 DAI to 42 DAI) (Fig. 2). Therefore, compared to the strain PYN-infected periwinkle, the earlier leaf symptom appearance and more severe inhibition of new flush growth observed in strain PGD-infected periwinkle indicated strain PGD could have a relatively stronger virulence than strain PYN in periwinkle.

### Temporal dynamic of two “*Ca*. Liberibacter asiaticus” strains and the associated phage in periwinkle.

**FIG 3:**
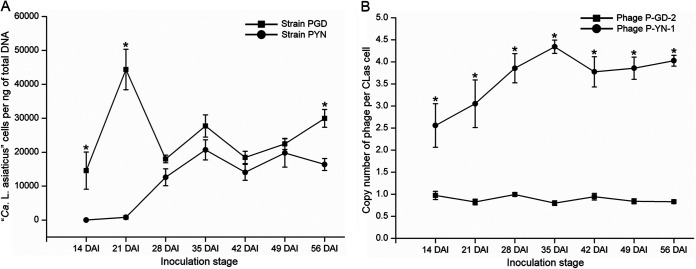
Quantification of “*Candidatus* Liberibacter asiaticus” and the associated phage in periwinkle (Catharanthus roseus) after inoculation. (A) “*Ca*. Liberibacter asiaticus” population; (B) phage copy number. DAI, days after inoculation. Single-factor analysis of variance (ANOVA) followed by Duncan’s multiple-comparison test at 95% (*P* < 0.05) confidence interval was used to determine statistical significance. *, *P* < 0.05.

Quantification analysis showed a different proliferation pattern between strain PGD and strain PYN in new flush of inoculated periwinkle before 28 DAI (Fig. 3A). The population of strain PGD in newly grown flushes of inoculated periwinkle rapidly increased, peaked at 21 DAI (44,389 ± 5,959 “*Ca*. Liberibacter asiaticus” cells per ng of total DNA), and then rapidly decreased at 28 DAI (Fig. 3A). However, the population of strain PYN was maintained in a lowest level in inoculated periwinkle at both 14 and 21 DAI, which was then followed by a rapid increase at 28 DAI (Fig. 3A). Statistical analysis showed that the bacterial concentration observed in new flush of strain PGD-infected periwinkle was significantly higher than those observed in strain PYN-infected periwinkle at 14 and 21 DAI (*P* < 0.05) (Fig. 3A), indicating a faster colonization and proliferation of strain PGD than strain PYN in the new flush at the early infection stage (before 21 DAI). The rapid colonization and higher population of strain PGD observed in new flush could be responsible for the earlier leaf symptom appearance and more severe inhibition in growth of new flush at earlier infection stages (Fig. 3A), In contrast, the slow increase of the strain PYN population in new flush before 21 DAI suggested a delayed infection of strain PYN in periwinkle at the early infection stage. After 28 DAI, strains PGD and PYN showed similar proliferation patterns in the new flush: i.e., the increase and decrease of the “*Ca*. Liberibacter asiaticus” population alternated between 35 DAI and 49 DAI (Fig. 3A). However, the concentration of strain PGD was higher than those of strain PYN in periwinkle at the same inoculation stage between 35 and 56 DAI, although the difference was mostly not significant (Fig. 3A).

PCR results showed that strain PGD harbored a type 2 phage (named P-GD-2) and strain PYN harbored a type 1 phage (named P-YN-1) (see Table S1 in the supplemental material). Phage copy number analysis showed that P-GD-2 phage was present as a single copy in strain PGD, while P-YN-1 phage was present as multiple copies (~2.5 to 4.3 copies) per “*Ca*. Liberibacter asiaticus” cell in strain PYN between 14 and 56 DAI (Fig. 3B). A previous study had shown that type 1 phage harbors the lytic cycle genes and its lytic cycle was strongly induced in infected periwinkle, with evidence of phage particles observed, while type 2 phage lacked the lysis-related genes and carried several genes involved in lysogenic conversion (12). Based on the presence of multiple copies of P-YN-1 phage (type 1 phage) and the low population of strain PYN in infected periwinkle at the early infection stage, P-YN-1 phage may undergo a lytic cycle in strain PYN. In contrast, the stable single copy of P-GD-2 phage observed in strain PGD suggested that P-GD-2 phage could be integrated into the strain PGD genome as the prophage form.

However, whether the interactions between strains PGD and PYN and type 1 and type 2 phages were related to the different symptom development in infected periwinkle remains unclear. To further reveal the possible mechanism of difference in symptom development and proliferation pattern between strain PGD and strain PYN in periwinkle, comparative genomic and transcriptomic analyses of the two strains in periwinkle were conducted.

### Genome comparison of “*Ca*. Liberibacter asiaticus” strains PGD and PYN.

Genome sequencing generated totals of 52,891,108 and 47,471,006 reads from periwinkle leaf midribs infected with strains PGD and PYN, respectively. Read mapping identified totals of 813,324 and 686,031 “*Ca*. Liberibacter asiaticus” reads in strain PGD and PYN samples, respectively. HiSeq reads mapping to three types of phage sequences (SC1, SC2, and P-JXGC-3) showed that strain PGD harbored a type 2 phage (P-GD-2), while strain PYN harbored a type 1 phage (P-YN-1) (see Fig. S1 in the supplemental material). The draft genome sequence of strain PGD (accession no. CP100754) comprised a total of 1,230,220 bp (99.17×, with G+C content of 36.41%) and harbored a type 2 prophage (37,789 bp, from bp 1191944 to 1229732, namely, P-GD-2). The genome sequence of strain PYN (CP100417) contained a total of 1,231,257 bp (83.58 ×, with G+C content of 36.48%) and harbored a type 1 phage/prophage (39,023 bp, from bp 1191938 to 1230960, namely, P-YN-1). The annotation result showed that strain PGD had 1050 open reading frames (ORFs) and strain PYN had 1048 ORFs. Genome comparison identified a total of 1034 genes from strain PGD as homologous with strain PYN, while a total of 1033 genes from strain PYN were homologous with strain PGD. Strain PGD contained 16 unique genes, and strain PYN harbored 15 unique genes (Table 1). Among strain-unique genes, 12 unique genes of strain PGD and 11 unique genes of strain PYN were located at the prophage region (Table 1; Fig. S2), while 4 unique genes of each strain were located at the chromosomal region (Table 1).

**TABLE 1:** General information and expression level of unique genes of “*Ca.* Liberibacter asiaticus” strains PGD and PYN

Gene no.	Locus tag	TPM	Position		Length (bp)	Region	Product
			Start	End			
PGD unique genes							
1	NKF51_00465	0	101306	101599	294	Chromosomal	Hypothetical protein
2	NKF51_04800	706	105375	106523	1,149	Chromosomal	Hypothetical protein
3	NKF51_04805	237	1080073	1080354	282	Chromosomal	Hypothetical protein
4	NKF51_04810	409	1080351	1080677	327	Chromosomal	Hypothetical protein
5	NKF51_05355	0	1195711	1195875	165	Prophage	Coiled coil fibrinogen
6	NKF51_05360	672	1196191	1197135	945	Prophage	Phage-related protein
7	NKF51_05365	0	1197156	1197632	477	Prophage	Phage-related protein
8	NKF51_05380	495	1199075	1201507	2,433	Prophage	Phage structural protein
9	NKF51_05385	191	1201504	1202727	1,224	Prophage	Phage-related protein
10	NKF51_05390	650	1202711	1204972	2,262	Prophage	Phage-related protein
11	NKF51_05395	1,436	1204974	1205462	489	Prophage	Integrase
12	NKF51_05400	1,299	1205464	1207188	1,725	Prophage	Exodeoxyribonuclease
13	NKF51_05405	1,245	1207195	1208967	1,773	Prophage	Phage-related protein
14	NKF51_05410	454	1208960	1209475	516	Prophage	Phage major tail subunit protein
15	NKF51_05425	0	1211271	1211543	273	Prophage	Phage-related glutathione peroxidase
16	NKF51_05430	599	1211540	1213159	1,620	Prophage	Phage-related head-to-tail joining protein
PYN unique genes							
1	NLY32_02375	0	530438	530632	195	Chromosomal	Hypothetical protein
2	NLY32_04415	0	992133	992318	186	Chromosomal	Hypothetical protein
3	NLY32_04805	0	1079732	1080379	648	Chromosomal	Hypothetical protein
4	NLY32_04810	0	1080352	1080930	579	Chromosomal	Hypothetical protein
5	NLY32_05360	691	1197385	1199979	2,595	Prophage	Phage structural protein
6	NLY32_05365	883	1199976	1201412	1,437	Prophage	Endolysin
7	NLY32_05370	551	1201430	1203493	2,064	Prophage	Phage-related protein
8	NLY32_05375	593	1203550	1204065	516	Prophage	Phage-related protein
9	NLY32_05380	746	1204046	1208089	4,044	Prophage	Cell envelope integrity protein TolA
10	NLY32_05385	881	1208114	1209850	1,737	Prophage	Phage-related protein
11	NLY32_05390	1,822	1209843	1210370	528	Prophage	Phage major tail subunit protein
12	NLY32_05405	265	1212074	1212403	330	Prophage	Phage-related glutathione peroxidase
13	NLY32_05410	471	1212396	1214066	1,671	Prophage	Phage-related head-to-tail joining protein
14	NLY32_05415	263	1214063	1214395	333	Prophage	Holin
15	NLY32_05420	1,129	1214392	1214817	426	Prophage	Hypothetical protein

It was found that the 12 prophage genes unique to strain PGD were mainly involved in phage lysogenic conversion, structural proteins, and oxidative stress resistance, including genes coding for an integrase (NKF51_05395), an exodeoxyribonuclease (NKF51_05400), a coiled-coil fibrinogen or tail needle protein (NKF51_05355), a phage major tail subunit protein (NKF51_05410), a phage-related glutathione peroxidase (NKF51_05425), a head-to-tail joining protein (NKF51_05430), a structural protein (NKF51_05380), and 5 phage-related proteins with unknown function (Table 1). In contrast, 11 prophage genes unique to strain PYN were mainly involved in the phage lytic cycle, antibacterial function, structural proteins, and oxidative stress resistance, including coding for an endolysin (NLY32_05365), a holin (NLY32_05415), a colicin (NLY32_05380), a phage structural protein (NLY32_05360), a phage major tail subunit protein (NLY32_05390), a head-to-tail joining protein (NLY32_05410), a phage-related glutathione peroxidase (NLY32_05405), and 4 phage-related hypothetical proteins. In addition, all chromosomal unique genes of strain PGD/strain PYN were annotated with unknown function (Table 1).

### Genome-wide gene expression profiling of two “*Ca*. Liberibacter asiaticus” strains in periwinkle.

**FIG 4:**
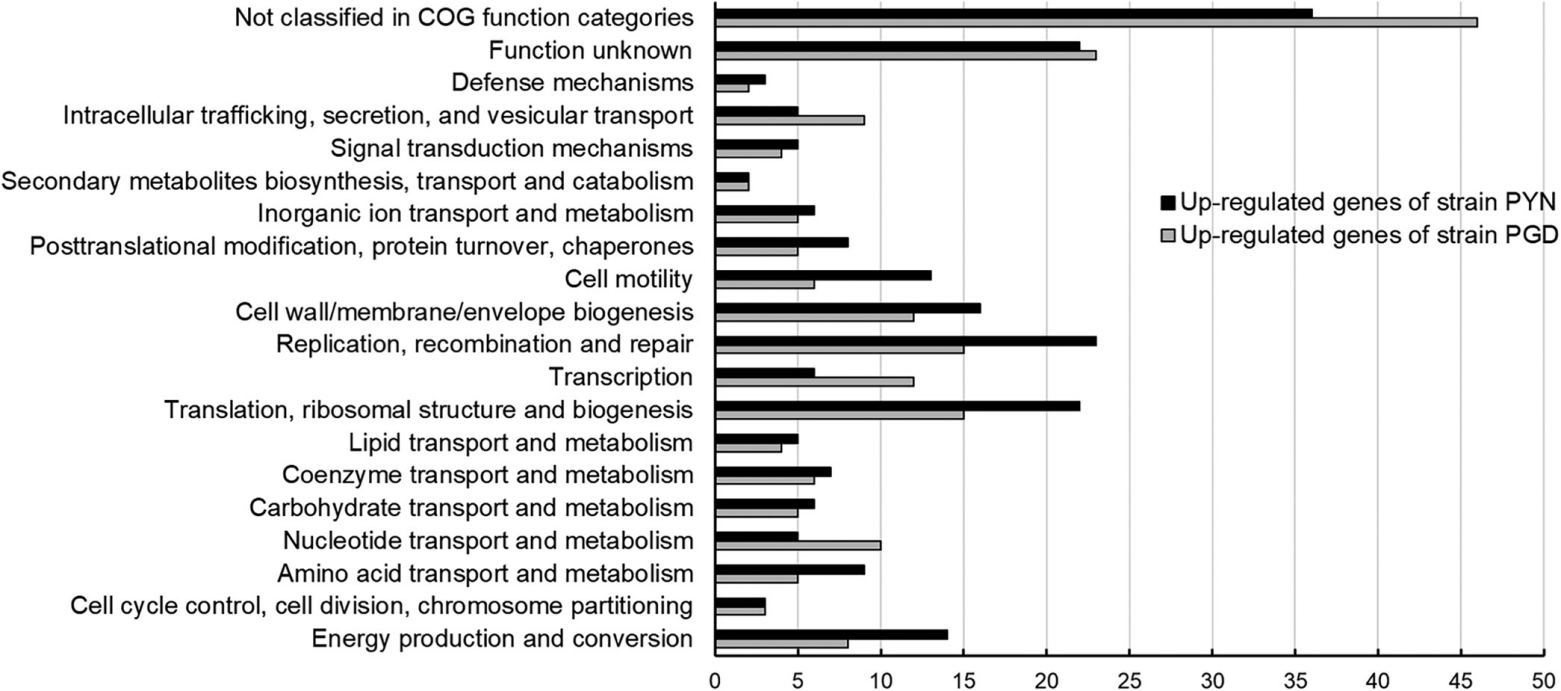
Functional classification of differentially expressed genes (DEGs) between “*Candidatus* Liberibacter asiaticus” strains PGD and PYN at 35 DAI. A log_2_ fold change of >|1| and *P* value of <0.05 were set as cutoff values to identify DEGs.

Approximately 127 and 101 million HiSeq reads were generated from strain PGD-infected and PYN-infected periwinkle leaf midribs (35 DAI), respectively. Reference-based mapping identified a total of 40,874 “*Ca*. Liberibacter asiaticus” reads from strain PGD transcriptome sequencing (RNA-Seq) data and 31,401 “*Ca*. Liberibacter asiaticus” reads from strain PYN RNA-Seq data. Transcriptomic analyses identified a total of 413 differentially expressed genes (DEGs) between strains PGD and PYN (log_2_ fold change of >|1| and *P* < 0.05). Of 413 DEGs, 197 were significantly upregulated in strain PGD and 216 were significantly upregulated in strain PYN. Function classification of 413 DEGs identified a total of 19 functional groups and 1 not classified in the COG functional categories (Fig. 4). A large number of DEGs involved in replication, recombination, and repair, translation, ribosomal structure and biogenesis, cell wall/membrane/envelope biogenesis, energy production and conversion, and cell motility were upregulated in either strain PGD or strain PYN, while more DEGs involved in the categories described above were found in strain PYN than strain PGD (Fig. 4). In contrast, a higher number of upregulated genes involved in transcription, nucleotide transport and metabolism, intracellular trafficking, secretion, and vesicular transport were identified in strain PGD than those observed in strain PYN (Fig. 4). The categories of DEGs and strain-unique genes related to difference in pathogenicity and bacterial proliferation between two “*Ca*. Liberibacter asiaticus” strains in periwinkle are described in detail in the following sections.

**(i) Phage/prophage genes.** Genome sequence analysis showed that strain PGD harbored a type 2 phage (P-GD-2) and strain PYN harbored a type 1 phage (P-YN-1) (Fig. S1). Genes from type 1 or 2 phage or prophage had been found to influence “*Ca*. Liberibacter asiaticus” biology and pathogenicity (14, 27–29). Therefore, the global gene expression level of two phages, including phage type-unique and homologous genes, were compared and analyzed in this study.

Among the 12 unique genes of prophage P-GD-1, three genes, including genes coding for an integrase (NKF51_05395 [TPM = 1,436]) (TPM is transcripts per kilobase per million), an exodeoxyribonuclease (NKF51_05400 [TPM = 1,299]), and a phage-related protein (NKF51_05405 [TPM = 1,245]), were highly expressed in strain PGD-infected periwinkle (Table 1). Phage integrase is a key enzyme involved in the lysogenic cycle during phage infection through mediation of unidirectional site-specific recombination between phage and bacterial genome (31). In addition, the phage-encoded exodeoxyribonuclease is essential for phage DNA replication and necessary for host DNA degradation and phage genetic recombination (32). The unique induction of genes involved in phage lysogenic conversation and recombination in strain PGD could facilitate the integration of the P-GD-2 phage genome into the “*Ca*. Liberibacter asiaticus” chromosome.

**FIG 5:**
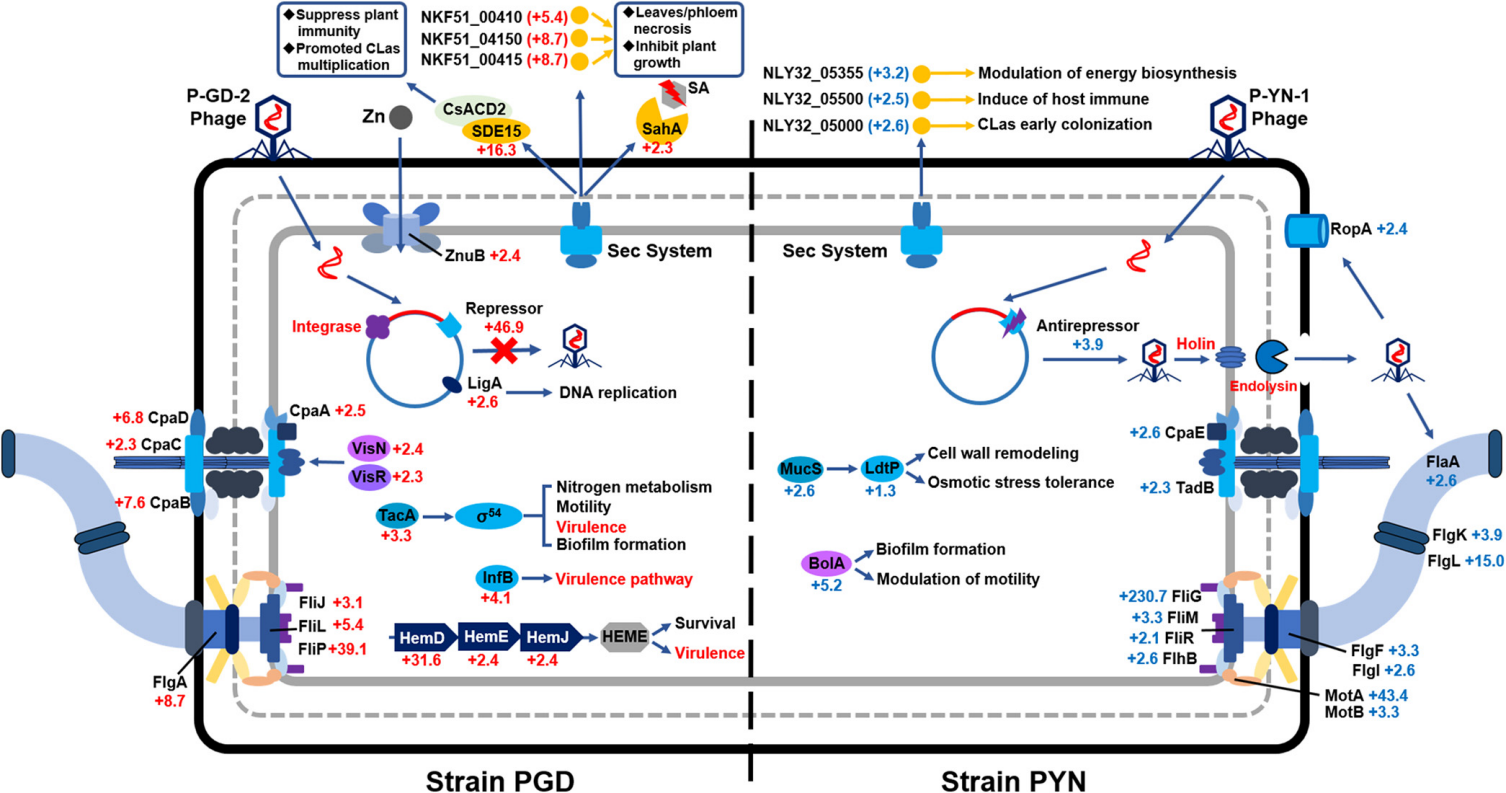
Selective regulation of “*Candidatus* Liberibacter asiaticus” genes involved in pathogenicity variation between two strains. Values indicated fold change of genes expression between strain PGD and strain PYN. A red number with a red plus sign represents upregulation in strain PGD, and a blue number with a blue plus sign represents upregulation in strain PYN.

In contrast, expression of phage P-YN-1 genes involved in the phage lytic cycle was detected in strain PYN-infected plants. The genes included those coding for an endolysin (NLY32_05365 [TPM = 883]) and a holin (NLY32_05415 [TPM = 263]) (Table 1 and Fig. 5). Both phage endolysin and holin played important roles in host bacterial cell lysis process (33, 34). Endolysins could break down host bacterial peptidoglycan at the terminal stage of the phage reproduction cycle, while holins could mediate lysis action by transporting endolysin across the cytoplasmic membrane (33, 34). A previous study found that the endolysin and holin encoded by type 1 phage may limit the host range and culturability of “*Ca*. Liberibacter asiaticus” (27). In addition, a phage major tail subunit protein (NLY32_05390 [TPM = 1,882]) that was unique to P-YN-1 phage was highly expressed in strain PYN-infected periwinkle (Table 1), while most phage-related structural proteins either were not expressed or were expressed at a very low level (TPM < 500) in strain PGD-infected periwinkle (Table 1). The unique expression of genes involved in the phage lytic cycle and assembly process indicated that the P-YN-1 phage may undergo a lytic cycle in strain PYN-infected periwinkle at the early infection stage.

In addition to phage type-unique genes, the levels of expression of homologous prophage genes between two “*Ca*. Liberibacter asiaticus” strains were also compared (Table S2). Sequence comparison identified a total of 31 prophage genes that were homologous between strains PGD and PYN (Table S2), while 16 of them were found to be differentially expressed between strains PGD and PYN in periwinkle (Table S2). Of 16 DEGs, 9 were upregulated in strain PGD, including genes coding for a DEAD/DEAH box helicase (NKF51_05320), a DNA ligase (NKF51_05325), a guanylate kinase (NKF51_05330), a phage-related helix-turn-helix repressor, C2 (NKF51_05445), a transferase (NKF51_05465), and 4 phage-related hypothetical proteins (NKF51_05350, NKF51_05370, NKF51_05455, and NKF51_05490) (Table S2). In contrast, seven prophage DEGs were upregulated in strain PYN, including genes coding for an autotransporter protein, LasAII/LasAI (NLY32_05355), a nonclassical secretory protein (NLY32_05500), an acetyltransferase (NLY32_05460), a Bro-N family phage antirepressor (NLY32_05505), an endonuclease (NKF32_05520), and two hypothetical proteins (NLY32_05485 and NLY32_05495) (Table S2). Among these DEGs, phage-carried repressor and antirepressor genes were found to play an important role in phage lytic-lysogenic switch (35–37). A previous study found that phage-encoded helix-turn-helix repressor C2 can help direct phage into the lysogenic cycle and allow the phage to reside inactively in the host bacterial chromosome (35). However, the antirepressors were mainly involved in induction of the phage lytic cycle by inactivation of repressors (36). In this study, the phage-related helix-turn-helix repressor C2 (NKF51_05445 [46.9-fold]) was only expressed in strain PGD-infected periwinkle, while the Bro-N family antirepressor (NLY32_05505) was significantly upregulated (3.2-fold) in strain PYN-infected periwinkle (Table S2). Based on the phage concentration data and the differential gene expression data, we propose that the P-YN-1 phage could exhibit lytic activity and limit the proliferation of strain PYN at the early “*Ca*. Liberibacter asiaticus” infection stage, while P-GD-2 phage may reside within “*Ca*. Liberibacter asiaticus” strain PGD’s genome as a prophage.

**(ii) Pathogenetic effectors.** Bacterial secretory proteins, called effectors, play critical roles in bacterial pathogenesis and bacterial-host interaction. Over 150 “*Ca*. Liberibacter asiaticus” effectors had been predicted in “*Ca*. Liberibacter asiaticus,” and some of them were functionally confirmed to be virulence factors or could induce or suppress the plant’s immune response by interacting with host proteins during infection (14, 29, 38–40). By selecting secretion-related Sec translocon-dependent extracytoplasmic proteins, a total of 36 effector genes were further selected for analyses in this study (Table S3). Of the 36 effector genes, nine were significantly upregulated in strain PGD-infected periwinkle. These included NKF51_03925, -04150, -00415, -00135, -03930, -03195, -00410, -04315, and -00200 (Fig. 5; Table S3). NKF51_03925 (16.3-fold) encoded a secreted protein, SDE15 (homologous to CLIBASIA_04025), which was previously found to suppress plant immunity by interaction with citrus protein CsACD2 and promoted “*Ca*. Liberibacter asiaticus” multiplication in citrus plants (41). Previous studies found that effector proteins encoded by NKF51_04150 (homologous to CLIBASIA_04250 [8.7-fold]), NKF51_00415 (homologous to CLIBASIA_00470 [8.7-fold]), and NKF51_00410 (homologous to CLIBASIA_00460 [5.4-fold]) were three virulence factors of “*Ca*. Liberibacter asiaticus” associated with necrosis in leaves/phloem and inhibition of plant growth (15, 42, 43). In addition, the upregulated NKF51_00200 gene (2.3-fold) in strain PGD-infected periwinkle encoded a salicylate hydroxylase (gene *sahA*, homologous to CLIBASIA_00255), which was previously found to degrade plant salicylic acid and suppressed plant defenses (44).

In contrast, eight effector-related genes were overexpressed in strain PYN-infected periwinkle compared to strain PGD-infected periwinkle, namely, NLY32_00170, -04440, -01565, -05000, -05500, -02235, -05355, and -00465 (Fig. 5; Table S3). NLY32_05000 (2.6-fold) is homologous to CLIBASIA_05115 in “*Ca*. Liberibacter asiaticus” strain Psy62, a recently identified effector (CaLasSDE115) that contributes to early colonization of “*Ca*. Liberibacter asiaticus” in citrus (45). NLY32_05355 (3.2-fold) encodes an autotransporter that has been found to target mitochondria and could be involved in modulation of plant energy biosynthesis (46). NLY32_05500 (2.5-fold) encodes a nonclassical secretory protein, which can induce strong immune responses in citrus (29). Other than NLY32_05000, -05355, and -05500, the functions of other upregulated effector-related DEGs in strain PYN-infected periwinkle have not been characterized yet, although some of them (NLY32_00170, -00465, -01565, -02235, and -04440) were experimentally validated to contain signal peptide genes (38).

Overall, compared to strain PYN-infected periwinkle, more upregulated effector genes involved in bacterial virulence and influence on the host immune system were identified in strain PGD-infected periwinkle. The difference in expression of effector-related genes between strains PGD and PYN in periwinkle indicated that the pathogenesis-related secretory effectors could play a critical role in determining the virulence variation between two strains.

**(iii) Transcriptional factors.** Bacterial transcriptional factors (TFs) are DNA-binding proteins that can regulate downstream gene expression by promoting or blocking the recruitment of RNA polymerase to specific genes (47). The TFs are global or local regulators of transcription initiation in bacteria, and some of them are related to bacterial pathogenicity by controlling the expression of virulence genes (47, 48). A total of 19 TF genes had been identified in “*Ca*. Liberibacter asiaticus” (49). In this study, 12 out of 19 TF genes were differentially expressed between strains PGD and PYN in periwinkle (Table S4). Among the 12 DEGs, eight were significantly upregulated in strain PGD-infected periwinkle, namely, NKF51_04080 (*rpoZ* [2.8-fold]), -02925 (*rpoH* [4.3-fold]), -02300 (*ihfB* [4.1-fold]), -01945 (*maf* [2.2-fold]), -03350 (*ftcR* [5.7-fold]), -02560 (*visN* [2.4-fold]), -02565 (*visR* [2.3-fold]), and -03965 (*tacA* [3.3-fold]) (Fig. 5; Table S4). The *rpoZ* and *rpoH* genes are involved in RNA polymerase assembly and RNA synthesis (50). The *tacA* gene is known as a highly conserved activator of σ^54^, a central regulator in many pathogenic bacteria, which has been linked to multiple cellular processes, such as nitrogen metabolism, motility, virulence, and biofilm formation (51). The *infB* gene is a key subunit of the integrated host factor (IHF), which was recently found to play an important role in regulation of multiple virulence pathways in the bacterial pathogen Dickeya zeae (52). Compared to strain PYN, the overexpression of TFs involved in regulation of bacterial virulence in strain PGD may contribute to the pathogenic process of strain PGD in periwinkle.

In contrast, four TF genes were induced in strain PYN-infected periwinkle, including NLY32_03060 (*bolA* [5.2-fold]), -04245 (*bolA* [3.9-fold]), -04930 (*marZ* [3.9-fold]), and -01115 (*mucS* [2.6-fold]) (Fig. 5; Table S4). *bolA* is present in most Gram-negative bacteria and plays important roles in biofilm development and bacterial motility, such as negatively modulating flagellar biosynthesis/swimming capacity and promoting biofilm formation (53). In addition, *mucS*, a member of the MarR family transcriptional regulators, and its regulated gene, *ldtP*, coding for l,d-transpeptidase, were found to be involved in “*Ca*. Liberibacter asiaticus” cell wall remodeling and mediating osmotic stress tolerance (54). Particularly, a recent study found that “*Ca*. Liberibacter asiaticus” *ldtP* was highly induced in “*Ca*. Liberibacter asiaticus” samples with a high abundance of phage and suggested that it may play an important role during phage infection by maintaining the envelope integrity of the “*Ca*. Liberibacter asiaticus” cell (55). The data in this work also showed that *ldtP* (NLY32_01110) was slightly upgraded (1.26-fold) in strain PYN-infected periwinkle compared to strain PGD-infected periwinkle (Table S4). This also indicated that the strain PYN could be under a relatively high phage pressure from lytic phage P-YN-1 in periwinkle.

**(iv) Cell surface components.** A total of 30 “*Ca*. Liberibacter asiaticus” DEGs involved in cell surface components (membrane, pilus, and flagella) were identified between strains PGD and PYN in periwinkle (Table S5). Seven out of 30 DEGs were related to “*Ca*. Liberibacter asiaticus” cell membrane, of which 6 were upregulated in strain PYN and only one was upregulated in strain PGD (Table S5). Six upregulated membrane-related DEGs in strain PYN were mainly involved in lipopolysaccharide (LPS) assembly, coding for outer or integral membrane proteins, and transport functions, including *ropA* (NLY32_00935), *lptD* (NLY32_01325), *inh* (NLY32_04635), *lolA* (NLY32_02020), *terC* (NLY32_01425), and *asmA* (NLY32_04165) (Fig. 5; Table S5). Particularly, the gene *ropA*, which encoded an essential outer membrane porin, was highly expressed in strain PYN (TPM = 3,438) and was significantly upregulated in strain PYN (2.4-fold) compared to strain PGD (Table S5). In addition to act as transport channels for multiple types of molecules, the bacterial porin encoded by *ropA* was also found to be utilized by the phage for cell adsorption and played a vital role in bacterium-phage interactions (56). The high expression of *rpoA* in strain PYN could be related to the infection of P-YN-1 phage. In strain PGD, only *murJ*, coding for an essential protein for peptidoglycan synthesis, was induced (Table S5).

Six DEGs involved in bacterial pilus biosynthesis were identified, including four Tad pilus-coding genes (*cpaA*, *cpaB*, *cpaC*, and *cpaD*) upregulated in strain PGD and two genes (*cpaE* and *tadB*) upregulated in strain PYN (Fig. 5; Table S5). “*Ca*. Liberibacter asiaticus” had a complete Tad pilus apparatus that was involved in cell adhesion (57). A previous study found that the Tad pili of *Liberibacter* were required for cell motility and DNA uptake (58). It was also found that the regulated genes of the “*Ca*. Liberibacter asiaticus” Tad pilus, *visNR* (59) (*visN*, 2.4-fold; *visR*, 2.3-fold), were also upregulated in strain PGD compared to strain PYN (Table S4). The induction of Tad pilus apparatus genes in strain PGD could promote its cell adhesion and movement in periwinkle (Fig. 5).

A total of 17 DEGs involved in flagellar biosynthesis were identified between two strains, including 6 upregulated in strain PGD and 11 upregulated in strain PYN (Table S5). In strain PGD, all six DEGs (*flgA*, *flgG*, *flgH*, *fliJ*, *fliL*, and *fliP*) were involved in the flagellar basal body (Table S5). However, DEGs involved in flagellar basal body (*flgF*, *flgI*, *fliG*, *fliM*, *fliR*, and *flhB*), flagellar filament (*flaA*), hook-wire junction (*flgK* and *flgL*), and flagellar rotation (*motA* and *motB*) were upregulated in strain PYN (Table S5). A previous study found that “*Ca*. Liberibacter asiaticus” movement in the phloem of citrus plant may not be mediated by flagella (60), which suggested that “*Ca*. Liberibacter asiaticus” flagella could have other functions besides movement. The bacterial flagella also acted as an important host receptor for phage adsorption in bacterial cells (61). Among flagellar components, the filament, formed by flagellin protein, was commonly targeted by the phage during adsorption (61, 62). The *flaA* gene was the only flagellin-encoding gene identified in the “*Ca*. Liberibacter asiaticus” genome (60). With a high density and multiple copies of phage P-YN-1 observed in strain PYN (Fig. 3B), the high expression level (NLY32_03325 [TPM = 1,094]) and the overexpression (2.6-fold) of *flaA* in strain PYN could contribute to the adsorption for P-YN-1 phage (Fig. 5).

**(v) Metabolic pathways.** Understanding the complex metabolic capabilities of “*Ca*. Liberibacter asiaticus” could help to unravel metabolic mechanisms associated with “*Ca*. Liberibacter asiaticus” growth, pathogenicity, and “*Ca*. Liberibacter asiaticus”-host interaction (63). In this study, DEGs between strains PGD and PYN were mainly involved in three metabolic pathways, including DNA replication, ABC transporters, and heme biosynthesis (Table S6).

Ten DEGs involved in the DNA replication pathway were identified between two strains, including seven upregulated in strain PGD and three upregulated in strain PYN (Table S6). Particularly, three DNA ligase *ligA* genes (NKF51_00010, -05220, and 05325) were upregulated in strain PGD (Table S6). NKF51_05220 encodes LigA. The protein contains a BRCT domain, which lacks the ligation activity but promotes protein-protein interaction (64). Sequence analysis showed that the BRCT-containing *ligA* gene (NKF51_05220) is located in the “*Ca*. Liberibacter asiaticus” chromosomal region, while the other two *ligA* genes (NKF51_00010 and -05325) were located in the prophage region of strain PGD. The prophage-derived DNA ligase can be used for virus metabolism, genome replication, recombination, and repair (65), as well as repair of host bacterial chromosome DNA double-strand breaks (66). Therefore, the upregulation of prophage-carried *ligA* could promote the bacterial DNA replication and proliferation of strain PGD in periwinkle.

A total of 13 DEGs related to the ABC transport system, which is mainly involved in uptake of nutrients/metabolites (amino acids and phosphates), enzyme cofactors (thiamine, iron/manganese, zinc) and lipids, were identified between two “*Ca*. Liberibacter asiaticus” strains (Table S6). Of the 13 DEGs, 9 were upregulated in strain PYN and 4 (*thiP*, *znuB*, *lolC*, and *mlaF*) were upregulated in strain PGD (Table S6). The gene *znuB* (NKF51_02430 [2.4-fold]), a member of the Znu system involved in zinc import, was significantly upregulated in strain PGD (Fig. 5; Table S6). Previous studies found that “*Ca*. Liberibacter asiaticus” had a functional high-affinity Znu system for zine uptake and the supplemental zinc in “*Ca*. Liberibacter asiaticus”-infected citrus could promote “*Ca*. Liberibacter asiaticus” replication but not reduce the zinc deficiency symptoms caused by “*Ca*. Liberibacter asiaticus” (67, 68), which indicated that the Znu system could be critical for survival and pathogenesis of “*Ca*. Liberibacter asiaticus” in citrus. The upregulation of *znuB* in strain PGD could contribute to “*Ca*. Liberibacter asiaticus” proliferation and symptom development in strain PGD-infected periwinkle. In addition, genes involved in ABC transporters of thiamine (NKF51_03175 [2.3-fold]), lipoprotein (NKF51_03730 [2.3-fold]), and phospholipid (NKF51_00050 [11.4-fold]) were also upregulated in strain PGD (Table S6). In strain PYN, the upregulated DEGs, including *thiQ*, *proV*, *proW*, *proX*, *lolD*, *sitA*, *pstA*, *aapJ*, and *aapQ*, were mainly involved in uptake of essential nutrients, including amino acids and phosphates (Table S6).

In heme biosynthesis, four DEGs were identified, including three genes (*hemD*, *hemE*, and *hemJ*) upregulated in strain PGD and only one (*hemH*) upregulated in strain PYN (Fig. 5; Table S6). The heme synthesized by bacteria not only served as an iron source for bacterial growth, but also served as a regulator involved in virulence regulation (69). In Pseudomonas aeruginosa, the *hemD* and *hemC* genes have been shown to be immediately downstream of *algR*, an essential transcriptional regulator for P. aeruginosa pathogenesis (70, 71). A previous study found that “*Ca*. Liberibacter asiaticus” heme biosynthesis was upregulated *in planta* compared to in psyllids and indicated the induction of heme biosynthesis genes may be important in survival and virulence of “*Ca*. Liberibacter asiaticus” *in planta* (72). Therefore, the activation of the heme biosynthesis pathway in strain PGD may not only be beneficial for “*Ca*. Liberibacter asiaticus” survival and colonization but also could be involved in the virulence of “*Ca*. Liberibacter asiaticus” in periwinkle.

### Conclusion.

In this study, we showed that two “*Ca*. Liberibacter asiaticus” strains harboring different types of phages exhibit differences in pathogenicity in periwinkle. Strain PGD (containing type 2 phage) exhibited higher virulence than strain PYN (containing type 1 phage) by exhibiting earlier leaf symptom and stronger inhibition in the growth of new flush. Compared to strain PYN, a faster proliferation of strain PGD was observed in inoculated periwinkle at the early infection stage. The possible activation of phage lytic cycle in strain PYN, as evidenced by unique expression of lytic cycle-related genes and multiple copies of phage, could limit the proliferation of strain PYN at the early infection stage and led to the delayed infection in periwinkle. In contrast, the high expression of genes involved in lysogenic conversion of phage P-GD-2 in strain PGD, as well as the observation of the single phage copy in strain PGD, indicated phage P-GD-1 could reside within the “*Ca*. Liberibacter asiaticus” genome as a prophage form. In addition, we also observed the significant differences in expression for many other genes, including pathogenetic secretory effectors, bacterial virulence-related transcriptional factors, the Znu transport system, and the heme biosynthesis pathway. We propose that the different activities of phages and differences in expression patterns of virulence factor genes may be two major determinants of virulence variation between the two “*Ca*. Liberibacter asiaticus” strains. The findings of this study provide new insight into the “*Ca*. Liberibacter asiaticus”-phage interaction and “*Ca*. Liberibacter asiaticus” pathogenicity.

## MATERIALS AND METHODS

### Bacterial strains.

“*Ca*. Liberibacter asiaticus” strain PYN, which contained the type 1 phage, was originally collected from HLB-affected Citrus limon cv. ‘Eureka’ (~5 years old) located in Ruili City (24°0′46.01′′N, 97°51′6.80′′E, 772 m) of Yunnan Province in December 2019. “*Ca*. Liberibacter asiaticus” strain PGD, which contained the type 2 phage, was originally collected from HLB-affected Citrus reticulata *Blanco* cv. ‘Shatangju’ (6 years old) located in Sihui City (23°19′38.75′′N, 112°44′2.54′′E, 12 m) of Guangdong Province in November 2019. All “*Ca*. Liberibacter asiaticus” strains were established and maintained in periwinkle according to a previous study (13). Briefly, “*Ca*. Liberibacter asiaticus”-infected buds that were collected from HLB-affected citrus trees in the field were moisturized immediately and brought back to the laboratory. All buds were tested with a “*Ca*. Liberibacter asiaticus” real-time PCR (73) and phage type-specific real-time PCR (22). The “*Ca*. Liberibacter asiaticus”-positive bud was then grafted onto healthy citrus (‘Shatangju’ mandarin or Eureka lemon) and transmitted to periwinkle [Catharanthus roseus (L.) G. Don.] via dodder (Cuscuta campestris Yunck). Dodder parasitized the citrus plants for a month and then attached to healthy periwinkle. Each of the “*Ca*. Liberibacter asiaticus” strains was then maintained and propagated in periwinkle through grafting after successful transmission from citrus to periwinkle.

### Periwinkle assay.

With the ability for rapid establishment and multiplication of “*Ca*. Liberibacter asiaticus” and shortened latency for symptom development, the periwinkle was selected as the indicator plant to evaluate the pathogenicity of “*Ca*. Liberibacter asiaticus” strains in this study. Healthy periwinkle plant was germinated from the seed and maintained in a greenhouse located at South China Agricultural University. The healthy periwinkle plant was ready for grafting only when it grew to about 20 cm. For each “*Ca*. Liberibacter asiaticus” strain, a total of 15 healthy periwinkle plants were used for top grafting with a single symptomatic leaf collected from “*Ca*. Liberibacter asiaticus”-infected periwinkle. Briefly, single leaves exhibiting yellowing/mottling symptoms were selected from periwinkle plants infected with each “*Ca*. Liberibacter asiaticus” strain as the grafting source. The top (~5 cm) of each healthy periwinkle plant was then cut off and grafted with a single symptomatic leaf. For the mock control, the “*Ca*. Liberibacter asiaticus”-free periwinkle leaf collected from healthy periwinkle was used for top grafting. All grafted periwinkle plants were fertilized with NPK water-soluble fertilizer weekly. Symptom development of “*Ca*. Liberibacter asiaticus”-inoculated periwinkle was recorded every 7 days. Leaf from new flush of each grafted periwinkle was sampled every 7 days from 14 DAI and immediately stored in liquid nitrogen for DNA and RNA extraction. Total RNA extracted from leaf samples collected at 35 DAI was selected for dual RNA-Seq. In addition, the length of newly grown flush from each grafted periwinkle was measured between 14 and 42 DAI.

### DNA and RNA extraction.

At each DAI, one leaf from each new growing flush was collected for DNA and RNA extraction. Only the leaf midribs were used for total plant DNA and RNA extraction. About 50 mg of leaf midribs tissue was used for total plant DNA extraction with the E.Z.N.A. high-performance plant DNA kit (Omega Bio-Tek, Doraville, GA, USA) according to the manufacturer’s instructions. For total plant RNA extraction, 100 mg of leaf midrib tissue was ground in liquid nitrogen and extraction was performed with the E.Z.N.A. plant RNA kit (Omega Bio-Tek) according to the manufacturer’s instructions. The quality of total plant DNA and RNA extracts was further evaluated by NanoDrop One (Thermo Fisher Scientific, Inc., Waltham, MA, USA). The concentration of total plant DNA and RNA was estimated using the Qubit 2.0 fluorometer (Thermo Fisher Scientific, Inc.). DNA and RNA preparations were stored at −80°C for further use.

### Quantification of “*Ca*. Liberibacter asiaticus” and the associated phage.

The quantification analysis of “*Ca*. Liberibacter asiaticus” was performed by TaqMan real-time PCR with primer sets (CLas4G/HLBr) and probes (HLBp) according to a previous study (73) (Table S7). Each quantitative PCR (qPCR) mixture contained 10 μL PerfectStart II probe qPCR SuperMix (TransGen Biotech, Beijing, China), 0.4 μL of each forward and reverse primer (10 μM), 0.2 μL TaqMan probe (5 μM), and 1 μL of DNA template (~25 ng) in a final volume of 20 μL, and the following procedure was performed: an initial denaturation step of 95°C for 2 min, followed by 40 cycles at 95°C for 10 s and 60°C for 30 s. Fluorescence signal was captured at the end of each 60°C step. All PCR assays were performed in the CFX Connect real-time system (Bio-Rad, CA, USA) with an automated baseline and threshold. The standard equation (*y* = −3.363*x* + 41.431) (*R*^2^ = 0.9995) for quantification of “*Ca*. Liberibacter asiaticus” was developed. Briefly, a recombinant plasmid, containing the pEASY-T1 cloning vector (TransGen Biotech, Beijing, China) and the CLas4G/HLBr primer region, was used for construction of the standard equation. The concentration of recombinant plasmid was determined by Qubit 2.0 (Thermo Fisher Scientific, Inc.). The copy number of the plasmid was calculated according to the following formula: no. of copies = (amt in ng × Avogadro no.)/(length in bp × 1 × 10^9^ × 650). A standard curve was generated from 10-fold serial dilutions of the recombinant plasmid DNA. The concentration of “*Ca*. Liberibacter asiaticus” was presented as “*Ca*. Liberibacter asiaticus” cells per nanogram of total DNA. The concentrations of two “*Ca*. Liberibacter asiaticus” strains in periwinkle leaf midribs at the same or different inoculation stages were analyzed by one-way analysis of variance (ANOVA), followed by Duncan’s multiple-comparison test (*P* < 0.05). The density of phage in “*Ca*. Liberibacter asiaticus” cells was indicated as copy number of phage per “*Ca*. Liberibacter asiaticus” cell by the threshold cycle (Δ*C_T_*) method with a “*Ca*. Liberibacter asiaticus”-specific primer set (CLas4G/HLBr) and phage type-specific primer set (type 1, SC1-045F/SC1-045R; type 2, SC2-035F/SC2-035R) according to a previous study (18): i.e., the relative copy number *R* = 2^−Δ^*^CT^*, where Δ*C_T_* = *C_T_* (SC1-045F/SC1-045R or SC2-035F/SC2-035R) − *C_T_* (primer set targeted single copy gene). The *C_T_* value of the primer set-targeted single-copy gene was converted from the *C_T_* value generated by primer set CLas4G/HLBr (targeted to three copies of the “*Ca*. Liberibacter asiaticus” 16S rRNA gene) with the equation *CT* (CLas4G/HLBr) + *X*, where *X* is 1.585.

### Genome sequencing and comparison.

For genome sequencing, DNA samples extracted from leaf midribs of periwinkle infected with the “*Ca*. Liberibacter asiaticus” PGD strain (35 DAI; *C_T_* = 18.01) or PYN strain (35 DAI; *C_T_* = 17.64) were selected. Genome sequencing was performed on the Illumina HiSeq 3000 platform (Illumina, Inc., San Diego, CA, USA) with 150-bp paired-end reads by a commercial sequencing company. The Illumina clean data were initially mapped to the *C*. *roseus* genome (JQHZ00000000.1) and *C*. *roseus* chloroplast genome (NC_021423.1) using Bowtie2 v.2.4.2 (74). The unmapped reads were retained for “*Ca*. Liberibacter asiaticus” genome assembly using CLC Genomics Workbench v.20.0 (Qiagen Bioinformatics, Aarhus, Denmark) with default parameters. For reference-based assembly, the strain A4 genome (CP010804.2), SC1 prophage (HQ377372.1), and SC2 prophage (HQ377373.1) were selected as the reference genomes (length fraction = 0.5 and similarity fraction = 0.8). Gap filling and prophage ligation were performed with *de novo* assembly contigs (minimum contig length = 500 bp) via BLAST+ v.2.10.0 (75). To more efficiently and systemically compare the genome sequences of strains PGD and PYN, as well as the associated phage sequence, the phage sequence was initially integrated into the “*Ca*. Liberibacter asiaticus” genome as the prophage sequence during sequence assembly. Genome annotation was performed with NCBI prokaryotic genome annotation pipeline. blastp and interlaced reference-based assembly were used to align annotated genes to identify homologous genes (>95% similarity) and strain-unique genes between two “*Ca*. Liberibacter asiaticus” strains.

### Transcriptome analyses.

For dual RNA-Seq, RNA samples extracted from periwinkle leaf midribs infected by the “*Ca*. Liberibacter asiaticus” PGD strain (average *C_T_* = 18.41) or PYN strain (average *C_T_* = 17.97) at 35 DAI were selected. For each “*Ca*. Liberibacter asiaticus” strain, three biological replicates of RNA samples (from different infected plants) were collected and used for RNA sequencing. Library preparation for dual RNA-Seq was performed with a TruSeq RNA library prep kit (Illumina, Inc., San Diego, CA, USA) by removing rRNA from total RNA. Sequencing was performed on the Illumina XTen platform (Illumina, Inc.) with 150-bp paired-end reads by a commercial sequencing company. For “*Ca*. Liberibacter asiaticus” transcriptome analysis, all clean reads from RNA libraries were mapped to the corresponding “*Ca*. Liberibacter asiaticus” genome by CLC Genomics Workbench v.20.0 (Qiagen Bioinformatics, Aarhus, Denmark) with default parameters. The number of unique reads mapped to each “*Ca*. Liberibacter asiaticus” gene was normalized to transcripts per kilobase per million (TPM): TPM = *A_i_* × 10^6^/sum(*A_1_* + *A_2_* + ··· + *A_i_*), where *A_i_* = total reads mapped to gene/gene base length. Determination of DEGs between strain PGD and strain PYN HiSeq data was performed with CLC Genomic Workbench v.20 based on the multifactorial statistics generalized linear model (GLM) with a cutoff setting of a log_2_ fold change of >|1| and *P* value of <0.05. All identified DEGs were functionally annotated using the eggNOG database (76). To validate the results of DEGs identified by RNA sequencing, seven “*Ca*. Liberibacter asiaticus” genes, including the salicylate hydroxylase gene *sahA* and six other secretion effector genes, were selected for reverse transcription-qPCR (RT-qPCR) (Table S7). The 16S rRNA genes of “*Ca*. Liberibacter asiaticus” were used as an internal reference and amplified using primer set CLas4G/HLBr (30). The comparative *C_T_* method (2^–ΔΔ^*^CT^*) was used to calculate the fold change of each gene from the qRT-PCR result. The log_2_ fold change was generated from the ratio of the relative expression value of strain PGD to that of strain PYN. For each gene, log_2_ fold change values of qRT-PCR were compared with those of the same gene from RNA-Seq analyses. The expression profiles of seven selected genes generated by RT-qPCR were consistent with RNA-Seq data, indicating the reliability of RNA-Seq analysis in this study (Fig. S3).

### Data availability.

All sequence reads generated in this project are available in the NCBI Short Read Archive under BioProject no. PRJNA854212. The genome sequences of the two “*Ca*. Liberibacter asiaticus” strains have been submitted to the GenBank database under accession no. CP100754.1 (strain PGD) and CP100417.1 (strain PYN).
