# Supplementary material for: Pathogenicity and Transcriptomic Analyses of Two “Candidatus Liberibacter asiaticus” Strains Harboring Different Types of Phages
Source: Microbiol Spectr. 2023 Apr 18;11(3):e00754-23. doi: 10.1128/spectrum.00754-23 (PMC10269750; doi:10.1128/spectrum.00754-23)
Supplement: Supplemental file 1 — Fig. S1 to S3 and Tables S1 to S7. Download spectrum.00754-23-s0001.pdf, PDF file, 0.6 MB [file spectrum.00754-23-s0001.pdf]

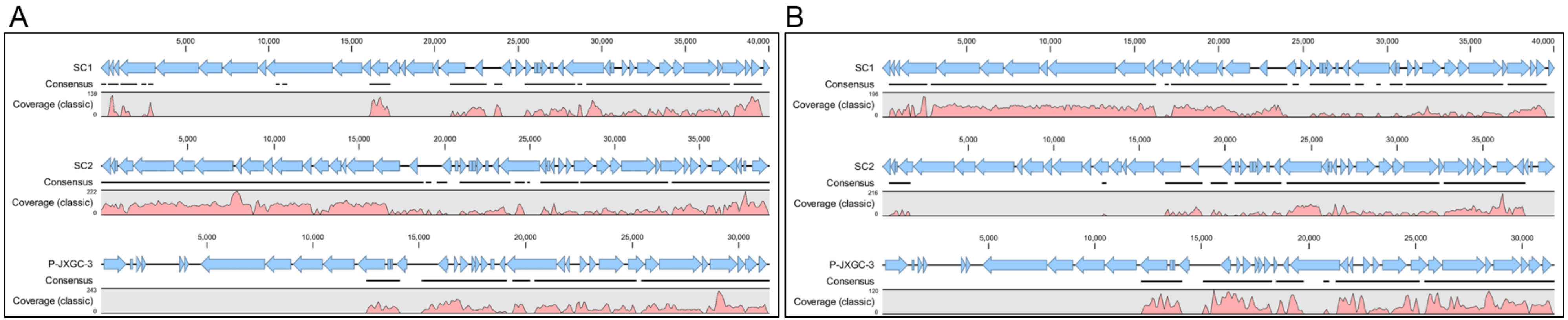

Fig. S1. Reads mapping to three phage sequences (SC1: HQ377372.1, SC2: HQ377373.1 and P-JXGC-3: KY661963.1) with HiSeq data of strain PGD (A) and PYN (B) infected periwinkle sample.

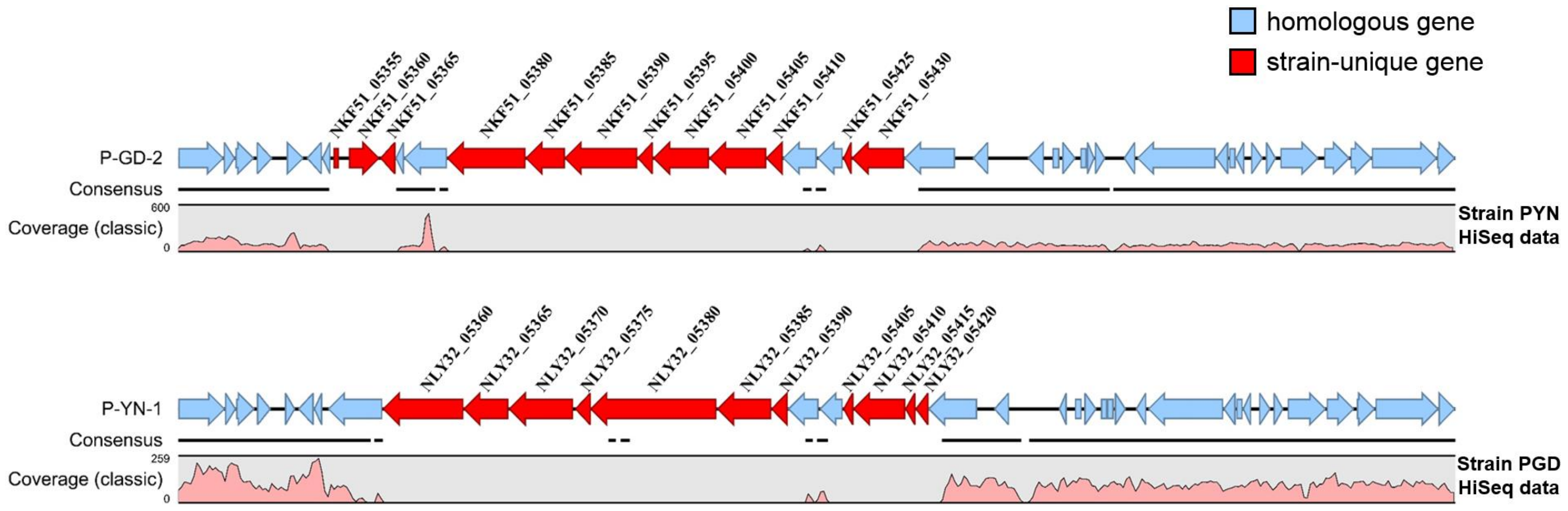

Fig. S2. Mapping track of "*Candidatus* Liberibacter asiaticus" strain PYN and strain PGD HiSeq data to phage P-GD-2 and P-YN-1 sequence. Code genes without reads coverage were identified as strain-unique genes.

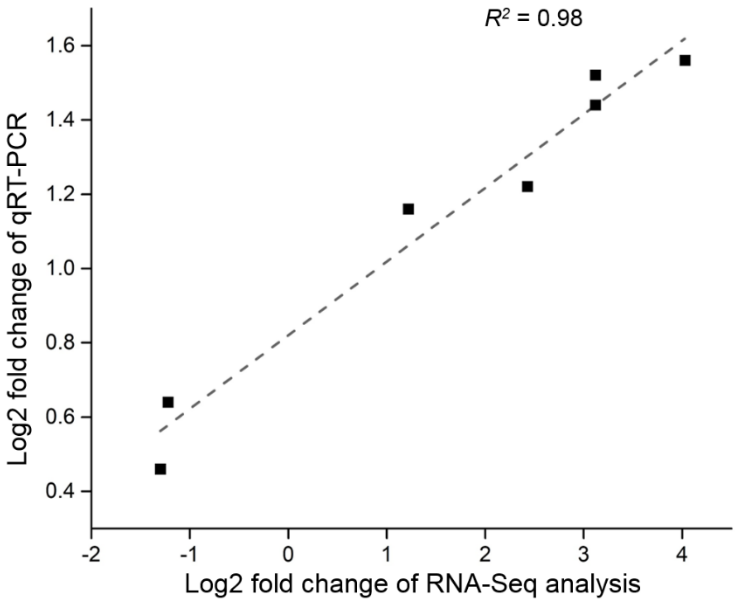

Fig. S3. Correlation of seven selected gene expression (Log2Fold Change) between RNA-seq and qPCR validation. The detail information of seven genes was listed in Table S7.

Table S1. Phage type-specific Real-time qPCR detection of two "*Candidatus* Liberibacter asiaticus" strains. NA, no amplification.

Strain	Ct value			
	CLas (CLas4G/HLBr)	Type 1 phage (SC1-045F/SC1-045R)	Type 2 (SC2-035F/SC2-035R)	Type 3 (PJXGC-8F/PJXGC-8R)
Strain PGD	18.01	NA	18.40	38.49
Strain PYN	17.64	15.66	NA	NA

Table S2. The homologous genes between phage/prophage P-GD-2 and P-YN-1.

No.	Strain PGD		Strain PYN		Identity %	Fold-change	Log2 fold-change	Product
	Locus tag	TPM	Locus tag	TPM				
1	NKF51_05320*	338	NLY32_05320	126	96	2.7	1.4	DEAD/DEAH box helicase
2	NKF51_05325*	1,393	NLY32_05325	0	100	100.4	6.7	DNA ligase, ligA
3	NKF51_05330*	117	NLY32_05330	0	100	16.3	4.0	Guanylate kinase
4	NKF51_05335	6,047	NLY32_05335	4,323	100	1.4	0.5	Trimeric autotransporter adhesin
5	NKF51_05340	13,931	NLY32_05340	26,335	67	1.9	-0.2	Collagen-like protein
6	NKF51_05345	1,904	NLY32_05345	1,199	96	1.7	0.8	Phage-related protein
7	NKF51_05350*	367	NLY32_05350	160	97	2.4	1.2	Phage-related protein
8	NKF51_05370*	367	NLY32_05350	160	99	2.4	1.2	Phage-related protein
9	NKF51_05375*	282	NLY32_05355	900	78	3.2	-1.8	Autotransporter protein
10	NKF51_05415	1,490	NLY32_05395	1,549	53	1.0	-0.1	Phage capsid protein
11	NKF51_05420	1,895	NLY32_05400	1,059	34	1.8	0.8	Reactive oxygen species-scavenging peroxidase
12	NKF51_05435	3,277	NLY32_05425	3,645	77	1.2	0.3	Terminase large subunit
13	NKF51_05440	2,393	NLY32_05430	2,951	100	1.2	-0.2	Phage terminase small subunit
14	NKF51_05445*	467	NLY32_05435	0	100	46.9	5.6	Phage-related repressor protein C2
15	NKF51_05450	0	NLY32_05440	0	93	1.0	0.0	Hypothetical protein
16	NKF51_05455*	3,998	NLY32_05445	1,558	97	2.6	1.4	Phage-related protein; PRK08999 superfamily
17	NKF51_05460	0	NLY32_05450	0	100	1.0	0.0	Phage-related UDP-glucuronosyltransferase
18	NKF51_05465*	138	NLY32_05455	0	100	138.1	7.1	Putative phage-related transferase
19	NKF51_05470*	0	NLY32_05460	131	100	9.4	-3.2	Phage-related acetyltransferase
20	NKF51_05475	3,876	NLY32_05465	3,364	90	1.4	0.5	Phage-related protein
21	NKF51_05480	507	NLY32_05470	627	87	1.2	-0.2	Phage/plasmid primase
22	NKF51_05485	343	NLY32_05475	561	96	1.6	-0.6	Phage-related protein

23	NKF51_05490*	162	NLY32_05480	0	100	8.7	3.1	Phage-related protein
24	NKF51_05495*	0	NLY32_05485	166	91	9.4	-3.2	Phage-related protein
25	NKF51_05500	270	NLY32_05490	353	98	1.3	-0.3	Phage-related protein
26	NKF51_05505*	0	NLY32_05495	135	100	9.4	-3.2	Phage-related protein
27	NKF51_05510*	258	NLY32_05500	637	88	2.5	-1.3	DUF2800 domain-containing protein
28	NKF51_05515*	42	NLY32_05505	166	95	3.9	-1.7	Bro-N family phage antirepressor
29	NKF51_05520	360	NLY32_05510	444	100	1.0	0.0	Phage-related helix-destabilizing protein
30	NKF51_05525	165	NLY32_05515	259	100	1.6	-0.5	DNA polymerase
31	NKF51_05530*	0	NLY32_05520	140	96	9.4	-3.2	Endonuclease

*, Differently expressing gene. Log2 fold change $\geqslant$ | 1 | and P-value < 0.05 was set as cutoff.

Table S3. Effector genes identified in CLas PGD and PYN strain and their relative expression.

No.	Locus_tag (Strain PGD/Strain PYN)	Gene length (bp)	TPM		Fold change	Log2 fold change	Product
			Strain PGD	Strain PYN			
1	NKF51_00135/NLY32_00140*	207	162	0	8.7	3.1	Hypothetical protein
2	NKF51_00165/NLY32_00170*	858	351	765	2.2	-1.1	Hypothetical protein
3	NKF51_00200/NLY32_00205*	1,143	819	383	2.3	1.2	Salicylate hydroxylase SahA
4	NKF51_00410/NLY32_00415*	297	788	147	5.4	2.4	Hypothetical protein
5	NKF51_00415/NLY32_00420*	156	214	0	8.7	3.1	Virulence factor
6	NKF51_00460/NLY32_00465*	294	0	149	9.5	-3.2	Hypothetical protein
7	NKF51_01035/NLY32_01035	471	355	371	1.0	0.1	Hypothetical protein
8	NKF51_01565/NLY32_01565*	426	157	411	2.6	-1.2	DUF1036 domain-containing protein
9	NKF51_02240/NLY32_02235*	489/471	1,436	4,179	2.9	-1.4	Hypothetical protein
10	NKF51_02305/NLY32_02300	882	5,042	4,017	1.4	0.5	Signal peptide peptidase, SppA
11	NKF51_02495/NLY32_02495	1,041	482	504	1.0	0.1	Substrate-binding domain-containing protein
12	NKF51_02530/NLY32_02530	1,470	523	982	1.9	-0.8	S1C family serine protease, Dop
13	NKF51_03030/NLY32_03030	816	1,475	1,501	1.1	0.1	Tetratricopeptide repeat protein
14	NKF51_03115/NLY32_03115	582	287	376	1.3	-0.3	Hypothetical protein
15	NKF51_03195/NLY32_03195*	363	92	0	8.7	3.1	Hypothetical protein
16	NKF51_03270/NLY32_03270	633	264	415	1.6	-0.5	DUF2155 domain-containing protein
17	NKF51_03605/NLY32_03605	342	1,857	2,302	1.2	-0.2	Hypothetical protein
18	NKF51_03925/NLY32_03925*	291	230	0	16.3	4.0	Secreted protein SDE15
19	NKF51_03930/NLY32_03930*	267	125	0	8.7	3.1	Hypothetical protein
20	NKF51_03935/NLY32_03935	480	0	0	0.0	0.0	Hypothetical protein
21	NKF51_04150/NLY32_04150*	150	223	0	8.7	3.1	Hypothetical protein
22	NKF51_04230/NLY32_04230	690	388	254	1.5	0.5	Hypothetical protein
23	NKF51_04305/NLY32_04305	366	639	836	1.3	-0.3	Hypothetical protein

24	NKF51_04315/NLY32_04315*	369	7,973	2,370	3.7	1.9	Hypothetical protein
25	NKF51_04435/NLY32_04440*	213	157	411	2.6	-1.2	Hypothetical protein
26	NKF51_04445/NLY32_04450	1,227	136	178	1.1	0.2	Virulence factor
27	NKF51_04465/NLY32_04470	588	910	744	1.5	0.6	Hypothetical protein
28	NKF51_04485/NLY32_04490	351	3,905	2,492	1.7	0.8	DUF5330 domain-containing protein
29	NKF51_05000/NLY32_05000*	558	180	470	2.6	-1.2	Secreted protein SDE115
30	NKF51_05020/NLY32_05020	681	2,798	4,174	1.5	-0.5	Virulence factor
31	NKF51_05145/NLY32_05145	465	2,732	1,693	1.8	0.8	Hypothetical protein
32	NKF51_05150/NLY32_05150	258	1,296	2,373	1.8	-0.7	Hypothetical protein
33	NKF51_05290/NLY32_05290	762	527	746	1.4	-0.4	Hypothetical protein
34	NKF51_05375/NLY32_05355*	1,185/1,653	282	900	3.2	-1.8	Autotransporter protein LasAII/LasAI
35	NKF51_05420/NLY32_05400	741/702	1,895	1,059	1.8	0.8	Reactive oxygen species-scavenging Peroxidase
36	NKF51_05510/NLY32_05500*	1,167	258	637	2.5	-1.3	DUF2800 domain-containing protein

*, Differently expressing gene. Log2 fold change ⩾ | 1 | and P-value < 0.05 was set as cutoff.

Table S4. Differentially expressed transcription factor genes between CLas strain PGD and strain PYN.

No.	Locus_tag (Strain PGD/Strain PYN)	Gene length (bp)	TPM		Fold change	Log2 fold change	Product
			Strain PGD	Strain PYN			
1	NKF51_04080/NLY32_04080	369	2,446	948	2.8	1.5	DNA-directed RNA polymerase subunit omega RpoZ
2	NKF51_02925/NLY32_02925	909	1,913	481	4.3	2.1	RNA polymerase sigma factor RpoH
3	NKF51_02300/NLY32_02295	303	1,655	433	4.1	2.0	Integration host factor subunit beta IhfB
4	NKF51_01945/NLY32_01945	600	1,505	729	2.2	1.1	Transcription factor Maf
5	NKF51_03350/NLY32_03350	672	1,095	195	5.7	2.5	Flagellar transcriptional regulator FtcR
6	NKF51_02560/NLY32_02560	741	767	354	2.4	1.2	Transcriptional regulator of flagellar regulon VisN
7	NKF51_02565/NLY32_02565	708	519	247	2.3	1.2	Transcriptional regulator of flagellar regulon VisR
8	NKF51_03965/NLY32_03965	1,449	461	151	3.3	1.7	Sigma 54-interacting transcriptional regulator TacA
9	NKF51_03060/NLY32_03060	306	109	572	5.2	-2.1	BolA family transcriptional regulator BolA
10	NKF51_04245/NLY32_04245	240	139	547	3.9	-1.7	BolA family transcriptional regulator BolA
11	NKF51_04930/NLY32_04930	438	382	1,498	3.9	-1.4	division/cell wall cluster transcriptional repressor MraZ
12	NKF51_01115/NLY32_01115	516	194	509	2.6	-1.2	MarR family transcriptional regulator MucS

Table S5. Differentially expressed genes associated with cell surface components between CLas strain PGD and strain PYN.

No.	Locus_tag (Strain PGD/Strain PYN)	Gene length (bp)	TPM		Fold change	Log2 fold change	Product
			Strain PGD	Strain PYN			
Cell membrane biosynthesis							
1	NKF51_02220 - NKF51_02225 / NLY32_02220	720 - 843 / 1496	1751	263	3.9	2.0	Murein biosynthesis integral membrane protein MurJ
2	NKF51_00935/NLY32_00935	1,056	1425	3438	2.4	-1.1	Porin RopA
3	NKF51_01325/NLY32_01325	2,289	175	611	3.5	-1.7	LPS assembly protein LptD
4	NKF51_04630/NLY32_04635	489	0	268	26.5	-4.7	Protease inhibitor Inh/omp19 family protein Inh
5	NKF51_02020/NLY32_02020	615	109	569	5.2	-2.2	Outer-membrane lipoprotein carrier protein LolA
6	NKF51_01425/NLY32_01425	1,572	596	1586	2.7	-1.3	TerC family protein TerC
7	NKF51_04165/NLY32_04165	1,143	59	246	4.2	-1.5	AsmA family protein AsmA
Flagellar assembly							
8	NKF51_01240/NLY32_01240	459	73	0	8.7	3.1	Flagellar basal body P-ring formation chaperone FlgA
9	NKF51_01245/NLY32_01245	789	551	0	100.4	6.7	Flagellar basal-body rod protein FlgG
10	NKF51_01225/NLY32_01225	717	93	0	16.3	4.0	Flagellar basal body L-ring protein FlgH
11	NKF51_03860/NLY32_03860	312	2036	701	3.1	1.7	Flagellar export protein FliJ
12	NKF51_01220/NLY32_01220	519	451	84	5.4	2.4	Flagellar basal body-associated FliL
13	NKF51_01215/NLY32_01215	741	226	0	39.1	5.3	Flagellar type III secretion system pore protein FliP
14	NKF51_03325/NLY32_03325	1,359	418	1094	2.6	-1.3	Flagellar FlaA
15	NKF51_03360/NLY32_03360	1,443	46	182	3.9	-1.8	Flagellar hook-associated protein FlgK
16	NKF51_03365/NLY32_03365	1,074	62	937	15.0	-3.7	Flagellar hook-associated family protein FlgL
17	NKF51_02590/NLY32_02590	873	0	250	43.4	-5.4	Flagellar motor stator protein MotA
18	NKF51_03335/NLY32_03335	1,032	130	424	3.3	-1.6	OmpA family protein MotB

19	NKF51_02595/NLY32_02595	732	91	299	3.3	-1.5	Flagellar basal-body rod protein FlgF
20	NKF51_01235/NLY32_01235	1,110	120	315	2.6	-1.2	Flagellar basal body P-ring protein FlgI
21	NKF51_02575/NLY32_02575	1,038	0	1138	230.7	-7.9	Flagellar motor switch protein FliG
22	NKF51_02585/NLY32_02585	957	210	686	3.3	-1.6	FliM/FliN family flagellar motor switch protein FliM
23	NKF51_03405/NLY32_03405	747	358	761	2.1	-1.1	Flagellar type III secretion system protein FliR
24	NKF51_02570/NLY32_02570	1,065	63	164	2.6	-1.2	Flagellar type III secretion system protein FlhB
Pilus assembly							
25	NKF51_02385/NLY32_02385	531	756	329	2.5	1.3	Prepilin peptidase CpaA
26	NKF51_02390/NLY32_02390	792	802	110	7.6	2.9	Flp pilus assembly protein CpaB
27	NKF51_02395/NLY32_02395	1,425	657	307	2.3	1.2	Type II and III secretion system protein CpaC
28	NKF51_02400/NLY32_02400	669	850	131	6.8	2.8	Pilus assembly protein CpaD
29	NKF51_02405/NLY32_02405	1,284	260	681	2.6	-1.3	AAA family ATPase CpaE
30	NKF51_02415/NLY32_02415	978	308	716	2.3	-1.1	Type II secretion system F family protein TadB

Table S6. Differentially expressed genes associated with metabolic pathways between CLas strain PGD and strain PYN.

No.	Locus_tag (Strain PGD/Strain PYN)	Gene length (bp)	TPM		Fold change	Log2 fold change	Product
			Strain PGD	Strain PYN			
DNA replication							
1	NKF51_01925/NLY32_01925	720	929	364	2.8	1.5	DNA polymerase III subunit epsilon DnaQ
2	NKF51_01615/NLY32_01615	1,158	953	378	2.7	1.5	DNA polymerase III subunit beta DnaN
3	NKF51_02030/NLY32_02030	444	301	99	3.1	1.6	Ribonuclease HI RnhA
4	NKF51_04775/NLY32_04780	630	265	69	3.9	2.0	Ribonuclease HII rnhB
5	NKF51_00010/NLY32_00015	360	1,207	121	11.4	3.5	DNA ligase LigA
6	NKF51_05220/NLY32_05220	2,196	335	139	2.6	1.4	NAD-dependent DNA ligase LigA
7	NKF51_05325/NLY32_05325	360	1,393	0	100.4	6.7	DNA ligase LigA
8	NKF51_02205/NLY32_02205	453	74	193	2.6	-4.2	DNA polymerase III subunit gamma/tau DnaX
9	NKF51_01990/NLY32_01990	1,038	0	337	69.1	-6.1	DNA polymerase III subunit delta HolA
10	NKF51_01305/NLY32_01305	426	471	1,129	2.4	-1.1	DNA polymerase III subunit chi HolC
ABC transporters							
11	NKF51_03175/NLY32_03175	1,608	291	136	2.3	1.2	Thiamine/thiamine pyrophosphate ABC transporter permease ThiP
12	NKF51_03180/NLY32_03180	660	51	199	3.9	-1.7	Thiamine ABC transporter ATP-binding protein ThiQ
13	NKF51_02430/NLY32_02430	783	854	391	2.4	1.2	Metal ABC transporter permease ZnuB
14	NKF51_01060/NLY32_01060	1,047	192	501	2.6	-1.5	ATP-binding cassette domain-containing protein ProV
15	NKF51_01065/NLY32_01065	846	158	775	4.9	-2.0	ABC transporter permease subunit ProW
16	NKF51_01070/NLY32_01070	930	827	2,304	2.8	-1.4	ABC transporter substrate-binding protein ProX
17	NKF51_03730/NLY32_03730	1,254	720	349	2.3	1.2	Lipoprotein-releasing ABC transporter permease subunit LolC
18	NKF51_03735/NLY32_03735	687	49	127	2.6	-1.2	ABC transporter ATP-binding protein LolD

19	NKF51_03290/NLY32_03290	885	869	2,076	2.4	-1.1	ABC transporter substrate-binding protein SitA
20	NKF51_02505/NLY32_02505	1,278	392	992	2.5	-1.2	Phosphate ABC transporter permease PstA
21	NKF51_00205/NLY32_00210	1,029	227	638	2.8	-1.3	Amino acid ABC transporter substrate-binding protein AapJ
22	NKF51_00210/NLY32_00215	1,197	196	621	3.2	-1.5	ABC transporter permease subunit AapQ
23	NKF51_00050/NLY32_00055	774	648	57	11.4	3.5	ATP-binding cassette domain-containing protein MlaF/YrbF
HEME biosynthesis							
24	NKF51_04580/NLY32_04585	699	191	0	31.6	5.0	Uroporphyrinogen-III synthase HemD
25	NKF51_01950/NLY32_01950	1,041	193	84	2.4	1.3	Uroporphyrinogen decarboxylase HemE
26	NKF51_01955/NLY32_01955	537	187	81	2.4	1.2	Protoporphyrinogen oxidase HemJ
27	NKF51_00370/NLY32_00375	1,032	162	678	4.2	-1.9	Ferrochelatase HemH

Table S7. Primers used in this study.

Target gene ID	Product	Primer	Sequence (5' - 3')	Size (bp)	Reference	Purpose
L22532.1	16S rRNA	CLas_4G<br>HLBr	AGTCGAGCGCGTATGCGAAT<br>GCGTTATCCCGTAGAAAAAGGTAG	78	Bao *et al.*, 2020	CLas detection
SC1_gp045	Putative phage-related protein	SC1-045F<br>SC1-045R	CCGTTCGTCTTTTGCCCATA<br>GCATTCTTCGCATCATCGGA	87	Zheng *et al.*, 2021	Type 1 phage detection
SC2_gp035	Putative phage-related protein	SC2-035F<br>SC2-035R	AGGTCACAAGGATTTAGCCCA<br>CTCCTAATCCCGCACCGATA	86	Zheng *et al.*, 2021	Type 2 phage detection
PJXGC_gp08	Type I restriction-modification system, specificity subunit S	PJXGC-8F<br>PJXGC-8R	CGGCGCTGAACTCTTGTATT<br>AAGGGCGTTGTTCTTGTCAC	85	Zheng *et al.*, 2021	Type 3 phage detection
NKF51_00200/<br>NLY32_00205	Salicylate hydroxylase SahA	PGD_200F<br>PGD_200R	GACTTTGTTTTCCGCTCTGG<br>CCCATAAATTCCTCCCCAGT	93	This study	RT-qPCR verification
NKF51_00410/<br>NLY32_00415	Secreted protein	PGD_410F<br>PGD_410R	GCCTCGTATTGCAACAAAATC<br>ACGGCGGAAGAAGATGAAG	99	This study	RT-qPCR verification
NKF51_00415/<br>NLY32_00420	Secreted protein	PGD_415F<br>PGD_415R	CTATGCTTTAACCGCCCAAA<br>TAGCGCCTTTCTCTTGCTTC	86	This study	RT-qPCR verification
NKF51_03925/<br>NLY32_03925	Secreted protein	PGD_3925F<br>PGD_3925R	TCCCAATAGACGACTCCCATA<br>GCCGGGTTGTGGTTCTATTA	98	This study	RT-qPCR verification
NKF51_04150/<br>NLY32_04150	Secreted protein	PGD_4150F<br>PGD_4150R	GGAACCGTATTAATGCTTGCT<br>TTTTTCTCCGCACCTTTTT	86	This study	RT-qPCR verification
NLY32_05000/<br>NKF51_05000	Secreted protein	PYN_5000F<br>PYN_5000R	ATCCGCCAAATCTAAACGTG<br>CGAATATCTGCCAAAGAATCA	81	This study	RT-qPCR verification
NLY32_05500/<br>NKF51_05510	Secreted protein	PYN_5500F<br>PYN_5500R	CCCATCAACCAACACAACTG<br>CTCATCTTCACACGCCTTCA	88	This study	RT-qPCR verification
